# Impact of Heat Treatment Parameters on the Plastic Properties of 6061 Aluminum Alloy

**DOI:** 10.3390/ma18081705

**Published:** 2025-04-09

**Authors:** Xiangdong Jia, Zhenyu Fan, Zhan Luo, Gang Hu, Hongyao Zhang

**Affiliations:** College of Mechanical and Electronic Engineering, Nanjing Forestry University, 159 Longpan Road, Nanjing 210037, China; 15895894901@163.com (Z.F.); 15285499794@163.com (Z.L.); 18055600204@163.com (G.H.); zhanghongyao01@163.com (H.Z.)

**Keywords:** 6061 aluminum alloy, solution treatment, aging treatment, mechanical properties

## Abstract

The 6061 aluminum alloy is extensively utilized in the production of aircraft components, valve parts, and maritime equipment, owing to its exceptional corrosion resistance, weldability, machinability, and anodic oxidation performance. This study investigates the effects of different heat treatment parameters on the mechanical properties of 6061 aluminum alloy. A series of orthogonal experiments were conducted, including quasi-static tensile tests using a QJBV212F-300KN universal testing machine following different solution and aging treatments. Scanning electron microscopy (SEM) was employed for microstructural characterization, revealing the mechanisms by which different heat treatment conditions impact the alloy’s mechanical properties. The test results indicate that the plasticity of 6061 aluminum alloy improves progressively within the temperature range of 510 °C to 540 °C. However, when the solution treatment temperature is elevated to 570 °C, significant grain coarsening occurs, leading to increased brittleness at the grain boundaries and reduced plasticity. Additionally, the elongation of 6061 aluminum alloy initially decreases and then increases as the aging time increases. Based on the experiments, a Hansel–Spittel constitutive model was developed, incorporating temperature, strain rate, and strain effects to accurately predict the flow stress of 6061 aluminum alloy under varying heat treatment conditions.

## 1. Introduction

Aluminum alloys are preferred for lightweight structural design because of their low density, lightweight nature, corrosion resistance, and high specific strength [1]. However, the mechanical properties of aluminum alloys are significantly influenced by their final heat treatment state and deformation conditions. As such, understanding the fundamental mechanical properties of aluminum alloys under various heat treatment and deformation conditions is a key area of current research [2]. Zhang et al. [3] investigated the effects of solution treatment and single-stage aging on the microstructure and properties of 7075 aluminum alloy, and developed precipitation kinetics equations under various aging conditions. Liu et al. [4] demonstrated that for 7075 aluminum alloy sheets, optimal stamping results are achieved with a solution treatment at 510 °C for 30 min followed by artificial aging at 120 °C for 24 h. Contact solution treatment (CST) increases the solubility of the second phase with higher contact temperatures and longer holding times, but prolonged high-temperature exposure causes coarsening of the dissolved second phase (primarily Mg_2_Si), reducing mechanical properties [5]. In addition, there are significant differences in the microstructure, mechanical properties, and corrosion resistance of extruded 7055 aluminum alloy under single-stage solution treatment (SST), enhanced solution treatment (EST), high-temperature pre-aging (HTPP), and multi-stage solution treatment (MST). HTPP improves the alloy’s corrosion resistance by modifying intergranular phases, but severely diminishes mechanical properties, whereas MST provides good corrosion resistance with minimal strength loss [6]. Compared to traditional multi-stage solution treatment, Lu et al. [7] observed that slow heating solution treatment can dissolve a larger amount of residual phases, significantly enhancing the hardness of the aluminum alloy while reducing its electrical conductivity. For 2219 aluminum alloy, as the heating rate increases or the solution treatment time is extended (30 to 70 min), the alloy’s hardness first increases and then decreases, while its electrical conductivity shows the opposite trend [8]. Gomes, R et al. investigates the effects of T5 and flash T6 heat treatments on blistering and mechanical properties of parts made from AlSi10MnMg and AlSi10Mg(Fe) alloys. T5 treatment eliminates blisters and improves strength but reduces ductility, with iron contamination affecting the secondary alloy’s properties [9]. The combination of enhanced solution treatment (EST) and non-isothermal aging (NIA) has a significant impact on the driving force for precipitate formation and the volume of precipitates in Al-Zn-Mg-Cu alloys [10]. Therefore, different heat treatment processes lead to distinct microstructures, resulting in varying macroscopic mechanical properties of aluminum alloys. Advanced characterization techniques such as scanning electron microscopy (SEM), energy dispersive spectroscopy (EDS), X-ray diffraction (XRD), differential scanning calorimetry (DSC), and electron backscatter diffraction (EBSD) are essential for accurately characterizing second phases, dislocations, and grain sizes, which are crucial for understanding the deformation mechanisms of these alloys [11].

The 6××× series aluminum alloys, which mainly contain magnesium (Mg) and silicon (Si) as their primary alloying elements, are commonly used in the transportation industry [12,13,14]. Among them, the 6061 aluminum alloy is particularly noted for its excellent corrosion resistance, weldability, workability, and anodizing properties, making it a preferred choice for producing aircraft components, valve parts, and marine accessories [15,16]. Although the strength of 6061 aluminum alloy is indeed lower than some specialized aviation alloys (such as 7075 or 2024), it still offers significant advantages in aircraft component applications. For instance, regarding environmental tolerance, the 6061-T6 alloy demonstrates a critical stress intensity factor (KISCC) of 28 MPa$\sqrt{m}$ in a 3.5% NaCl solution, which is significantly higher than that of 7075-T6 (approximately 10 MPa$\sqrt{m}$). This makes it particularly suitable for components exposed to high-humidity or salt-spray environments, such as the skin of maritime patrol aircraft [17]. The 6061 aluminum alloy differs from other series of aluminum alloys (such as 7075 or 2219 aluminum alloys), and therefore, its use in aircraft is different. Firstly, there are differences in alloy composition and strengthening mechanisms between 6061 aluminum alloy and 7075 or 2219 aluminum alloys. The 6061 alloy primarily contains magnesium (Mg) and silicon (Si) as its main alloying elements, achieving strengthening through the formation of Mg_2_Si precipitates. In contrast, the 7075 alloy is dominated by zinc (Zn) and magnesium (Mg), supplemented with copper (Cu), and relies on η-phase (MgZn_2_) for strengthening. The 2219 alloy, on the other hand, is copper (Cu)-based (approximately 6.3%), with its strengthening phase being the θ-phase (Al_2_Cu) [18]. Secondly, differences exist in the heat treatment parameters between 6061 and 7075 or 2219 alloys. For example, the typical process for 6061 involves solution treatment at 530–550 °C followed by artificial aging at 160–180 °C. In comparison, 7075 undergoes solution treatment at 470–490 °C and is then subjected to a multi-stage aging process [19]. Lastly, variations in precipitate behavior and microstructure are observed among these alloys. The 6061 alloy forms a sequence of metastable phases (e.g., β″ and β′) during aging, while 7075 focuses on controlling the distribution of η-phase to balance strength and corrosion resistance. The 2219 alloy emphasizes the thermal stability of θ-phase under extreme temperatures, critical for aerospace applications [20]. With the rapid advancement of manufacturing technologies, the performance requirements for 6061 aluminum alloys have continuously increased. Therefore, an in-depth study of the flow stress of 6061 aluminum alloy sheets is of great significance for optimizing plastic forming processes and improving forming quality. Arslankaya et al. [21] found that solution treatment and aging parameters significantly influence the elongation, yield strength, tensile strength, and hardness of 6061 aluminum alloy. Niu et al. [22] introduced a novel cryogenic treatment technique, optimizing process parameters through orthogonal experiments, which effectively reduce residual stress and improve the performance of the aluminum alloy. Zhang et al. [23] pointed out that when the solution treatment temperature of 6061 aluminum alloy exceeds 580 °C, overburning occurs, leading to a sharp decline in strength and ductility. Thus, it is critical to strictly control the solution treatment temperature to prevent overburning. In addition to the impact of heat treatment regimes on the plastic properties of 6061 aluminum alloy, deformation conditions such as deformation temperature and deformation rate also have a significant effect on its plastic performance parameters. Hot compression tests conducted using the Gleeble-3500 system (Dynamic Systems Inc., PA, American) at temperatures ranging from 350 °C to 510 °C and strain rates from 0.001 to 10 s^−1^ reveal that as deformation temperature increases and strain rate decreases, the uniform elongation of the aluminum alloy improves [24]. Although the maximum thermoplasticity was achieved at 500 °C, the thermoplasticity and superplasticity of 6061 aluminum alloy decreased with the increase in void volume fraction and its distribution [25]. Whether considering heat treatment regimes or deformation conditions, establishing a constitutive model under varying conditions is essential for optimizing the processing of 6061 aluminum alloy. Models such as Garofalo–Arrhenius, Fields–Backofe, Johnson–Cook, and Hansel–Spittel can be employed to construct the constitutive model of 6061 aluminum alloy [26]. However, discrepancies between the predictions of different theoretical models and experimental results are inevitable. Therefore, it is necessary to validate the accuracy of these models by calculating the correlation coefficient (R), mean absolute relative error (MARE), and root mean square deviation (RMSD) between the predicted and experimental values. This approach ensures the identification of the best-fitting model.

The aforementioned research findings indicate that the mechanical properties of 6061 aluminum alloy are largely influenced by its final heat treatment condition. To develop a reasonable plastic forming process, it is essential to clearly understand the mechanical property parameters of 6061 aluminum alloy under various heat treatment conditions. Achieving the maximum elongation while ensuring the strength requirements is crucial for optimizing the plastic forming process.

This study focuses on 6061 aluminum alloy cold-rolled thin plates. The mechanical properties under different heat treatment parameters were investigated through uniaxial tensile tests and microstructural analysis. The mechanisms by which varying heat treatment parameters affect the mechanical properties of 6061 aluminum alloy are analyzed, and a constitutive model for heat-treated 6061 aluminum alloy plates is developed based on this analysis.

## 2. Materials and Methods

The aluminum alloy used in this study is 6061 aluminum alloy cold-rolled plates. The elemental composition of 6061 aluminum alloy was analyzed using ICP-AES technology, as shown in Table 1. The tensile specimen was prepared by cutting the plates along three directions: the rolling direction (RD), at a 45° angle to the rolling direction (ST), and perpendicular to the rolling direction (LT). This approach was employed to investigate the influence of the rolling direction on the tensile properties of the aluminum alloy. The preparation of tensile specimens followed ASTM standards to ensure consistency and comparability of the samples.

**Figure 1:**
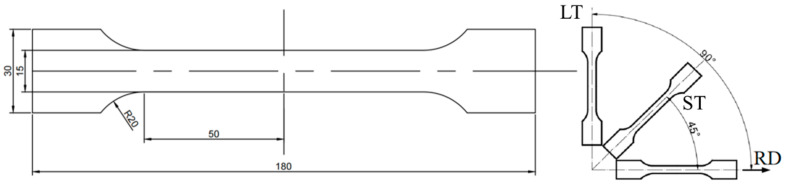
Sample size and cutting direction: (**left**) Sample shape and dimensions; (**right**) Three sampling layouts.

The dimensions and shape of the tensile specimens are illustrated in Figure 1. To minimize the impact of surface defects like cutting cracks and burrs, the samples were polished on both sides with sandpaper before testing.

**Figure 2:**
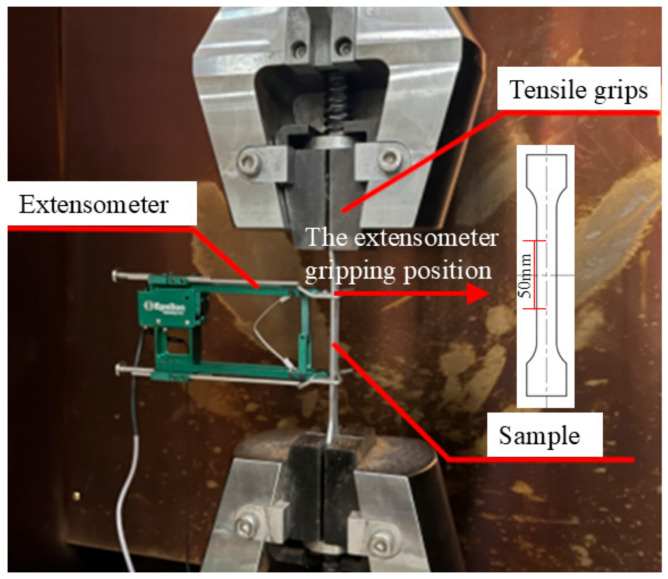
QJBV212F-300KN universal testing machine.

Quasi-static tensile tests were conducted at room temperature (25 °C) using a QJBV212F-300KN universal testing machine. The specimens with different rolling angles were tested at strain rates of 0.001 s^−1^, 0.01 s^−1^, and 0.05 s^−1^, as shown in Figure 2. The specimen is clamped at both ends, and the gauge length of the extensometer is 50 mm. The extensometer is attached to the gauge length of the specimen. The extensometer used in this study is produced by Epsilon (Epsilon Technology Corporation, Jackson, WY, USA), with the model being Axial Extensometer–Model 3542. After completing the tensile tests, samples were taken from the fracture surfaces of the specimens. The fracture surfaces were characterized using a Quanta 200 environmental scanning electron microscope (ESEM) (FEI Company, Hillsboro, OR, USA) to investigate the effects of different strain rates on the morphology of the tensile fractures.

**Figure 3:**
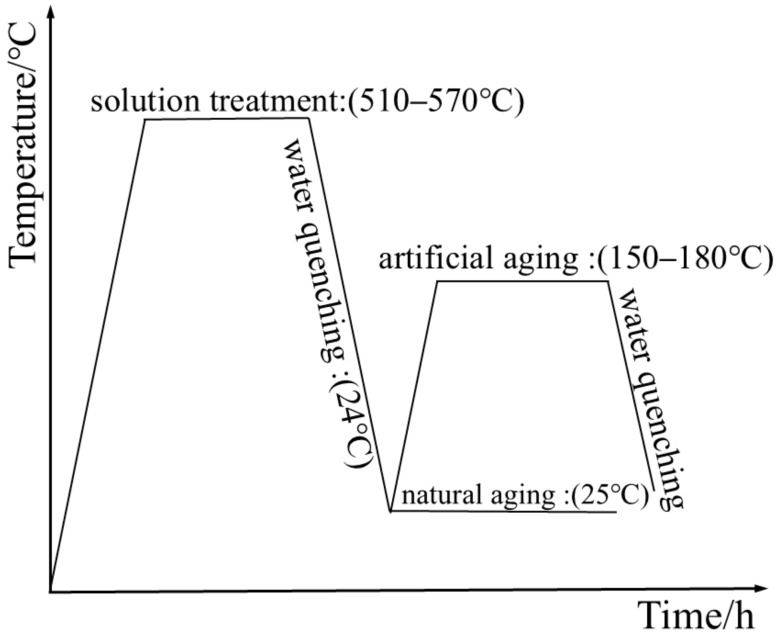
Heat treatment process diagram.

**Figure 4:**
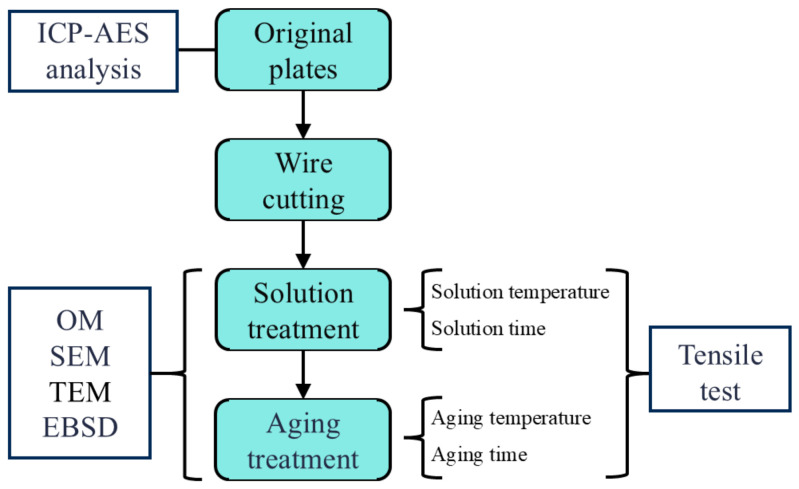
Test schematic diagram.

To further explore the effects of different heat treatment parameters on the properties of 6061 aluminum alloy, an orthogonal experiment was designed, as shown in Table 2. The process parameter values in this article are based on the heat treatment parameters of 6061 aluminum alloy T6. A parameter is taken as the solution temperature every 15 °C between 510 °C and 570 °C, and a parameter is taken as the aging temperature every 10 °C between 150 °C and 190 °C [27]. Initially, two identical specimens were subjected to solution treatment (510–570 °C) in a furnace at specific temperatures. After reaching the prescribed time, the specimens were removed and cooled using the same method (water cooling: 24 °C). One specimen was then subjected to artificial aging (150–190 °C) in the heating furnace, while the other was allowed to undergo natural aging at room temperature (25 °C). This setup was designed to compare the effects of natural aging and artificial aging, as depicted in Figure 3. All heat treatments were conducted in a KBF11Q-III (LabTech, Shanghai, China) box-type heat treatment furnace, with a temperature deviation of ±3 °C, a temperature display error of ±1 °C, and a heating rate of 15 °C/min. Finally, the aged specimens underwent quasi-static tensile testing. The schematic diagram of the test is shown in Figure 4.

## 3. Results and Discussion

### 3.1. The Effect of Strain Rate on Performance

**Figure 5:**
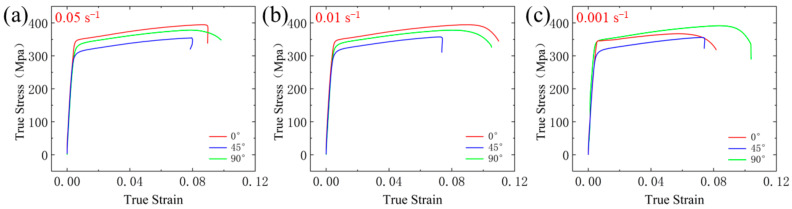
Stress–strain curves for RD, ST, and LT directions at different strain rates: (**a**) 0.05 s^−1^; (**b**) 0.01 s^−1^; (**c**) 0.001 s^−1^.

Strain rate is a crucial parameter in the mechanical properties of materials, significantly affecting the plastic behavior and strength of aluminum alloys. The true stress–strain curves of 6061 aluminum alloy under different strain rates are shown in Figure 5. The mechanical performance parameters are listed in Table 3.

From the experimental results, it can be observed that, in the initial deformation stage, the flow stress increases rapidly and linearly with strain. As the deformation progresses into the plastic stage, the rate of increase in stress slows down and approaches a steady-state flow. With further strain, the flow stress reaches a peak and then begins to decrease gradually, entering the necking and elongation stage. Finally, the necking region becomes unstable, and cracks initiate and propagate, leading to final fracture with a sharp drop in stress. Comparing the tensile test results along the rolling direction (RD), at a 45° angle to the rolling direction (ST), and perpendicular to the rolling direction (LT) under different strain rates, specimens oriented along the RD and LT directions exhibit similar mechanical properties. In contrast, specimens in the ST direction show distinct differences in mechanical performance compared to the other two directions. For example, at a strain rate of 0.05 s^−1^, the ST-direction specimens yield at a true stress of 306 MPa and fracture at a true stress of 355 MPa, with an elongation of only 7.9%, which is lower than the mechanical performance of RD- and LT-direction specimens under the same conditions. Similar trends were observed at strain rates of 0.01 s^−1^ and 0.001 s^−1^. The ST-direction specimens exhibit lower yield stress, tensile strength, and elongation compared to the RD- and LT-direction specimens. The mechanical performance parameters for RD and LT directions are quite similar, indicating that commercial cold-rolled 6061 aluminum plates exhibit significant anisotropy.

**Figure 6:**
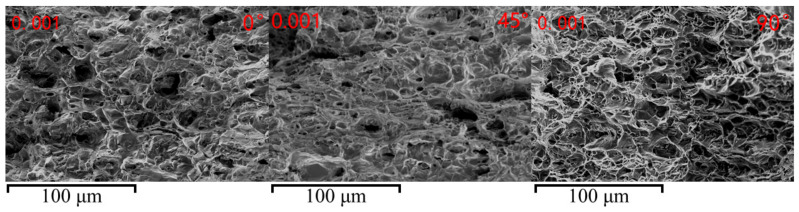
SEM images of fracture surfaces at 0.001 s^−1^.

The whole fracture surface was scanned, and it was found that the port morphology characteristics were evenly distributed, so the representative pictures were selected for analysis. Figure 6 is the SEM images of the fracture surfaces of tensile specimens tested at a strain rate of 0.001 s^−1^. The fracture characterization indicates that at this strain rate, 6061 aluminum alloy exhibits distinct ductile fracture characteristics. Comparing the fracture morphology of specimens cut in different directions, those cut along the rolling direction (RD) and perpendicular to the rolling direction (LT) show larger and deeper dimples compared to specimens cut at a 45° angle to the rolling direction (ST). In the samples along the RD and LT directions, dislocation slip and cross-slip are more likely to occur, which results in better material plasticity. In contrast, in the ST direction, dislocation movement is restricted, potentially leading to crack formation and, consequently, affecting plasticity. This suggests that the specimens in the RD and LT directions have better plasticity during deformation, which is consistent with the macro-mechanical performance results shown in Figure 5. Therefore, it can be concluded that 6061 aluminum alloy exhibits better plastic deformation characteristics along the rolling direction (RD) and perpendicular to the rolling direction (LT).

**Figure 7:**
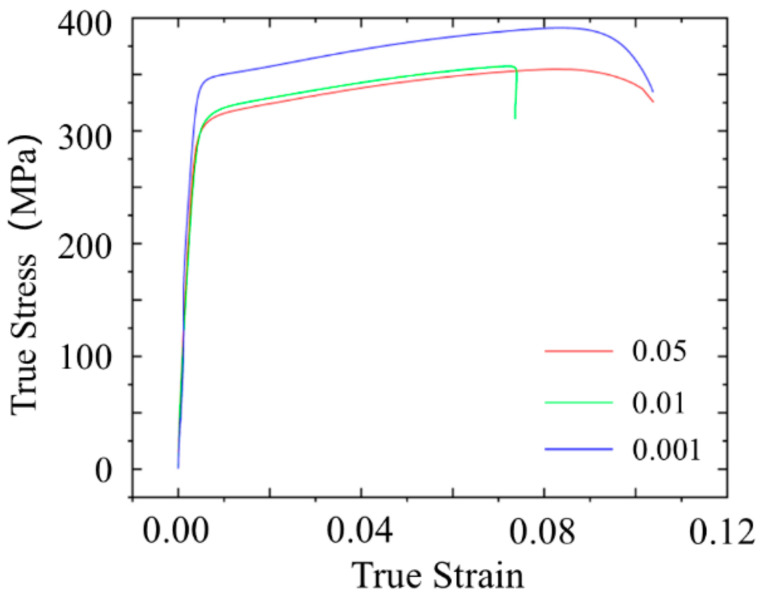
Stress–strain diagrams of tensile specimens under different strain rates.

The true stress–strain curves of 6061 aluminum alloy rolled perpendicular to the rolling direction (LT) at strain rates of 0.001 s^−1^, 0.01 s^−1^, and 0.05 s^−1^ are shown in Figure 7. The experimental results indicate that as the strain rate increases, both the yield strength and tensile strength of the 6061 aluminum alloy decrease. However, the plastic elongation rate initially decreases and then increases with the rise in strain rate. At a strain rate of 0.001 s^−1^, the alloy exhibits the maximum strength and elongation. The combination of sufficient time for dislocation recovery and uniform deformation leads to maximum strength and elongation at the lowest strain rate.

**Figure 8:**
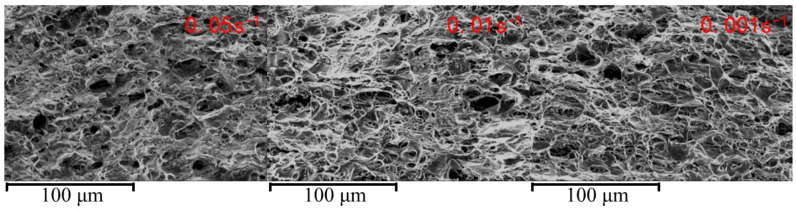
SEM images of fracture morphology under different strain rates.

The fracture morphology of the specimens under different strain rates, obtained via SEM, is shown in Figure 8. The SEM characterization reveals that, under all deformation conditions, the fractures of 6061 aluminum alloy exhibit distinct dimpled features. However, at a strain rate of 0.001 s^−1^, the dimples on the fracture surface are larger and deeper compared to those observed at strain rates of 0.05 s^−1^ and 0.01 s^−1^. Generally, larger and deeper dimples are indicative of better plasticity in the material, whereas finer dimples are typical of materials with slightly lower plasticity. At low strain rates (such as 0.001 s^−1^), dislocations in the material are more easily able to slip and undergo cross-slip. In this case, larger plastic deformation can be achieved through the continuous accumulation of dislocations, cross-slip, and the deformation of local grains. Due to the lower strain rate, dislocation motion faces less resistance, allowing the material to better absorb deformation energy, resulting in the formation of larger ductile pits and exhibiting improved plasticity. In contrast, at higher strain rates (such as 0.05 s^−1^ or 0.01 s^−1^), dislocation slip and cross-slip encounter more resistance, which limits the material’s plastic deformation. This leads to the formation of smaller and shallower ductile pits. At these higher strain rates, dislocations may not be able to release stress in a timely manner, increasing the likelihood of crack formation, thereby affecting the material’s plasticity. Thus, the 6061 aluminum alloy demonstrates the best plasticity at a strain rate of 0.001 s^−1^, which is consistent with the macro-mechanical performance test results.

In summary, for subsequent research aimed at investigating the effects of heat treatment parameters on the mechanical properties of 6061 aluminum alloy, specimens cut perpendicular to the rolling direction (LT) will be used as the research subjects. The mechanical performance parameters of 6061 aluminum alloy at a strain rate of 0.001 s^−1^ will serve as the evaluation criteria.

### 3.2. The Effect of Heat Treatment on Performance

Solution treatment and aging treatment are crucial heat treatment methods for adjusting the macro-mechanical properties of alloys by altering their microstructure. The primary objective of solution treatment is to dissolve solute elements into the alloy matrix, thereby enhancing the alloy’s plasticity and workability. Aging treatment, on the other hand, involves the formation of new strengthening phases or alteration of existing phases within the alloy to increase its strength and hardness while maintaining a certain level of plasticity. Therefore, for the 6061 aluminum alloy after solution and aging treatment, the influence of heat treatment parameters on its mechanical properties can be characterized by tensile test. In this paper, the unidirectional tensile test is used to evaluate the influence of heat treatment parameters on macroscopic mechanical properties.

**Figure 9:**
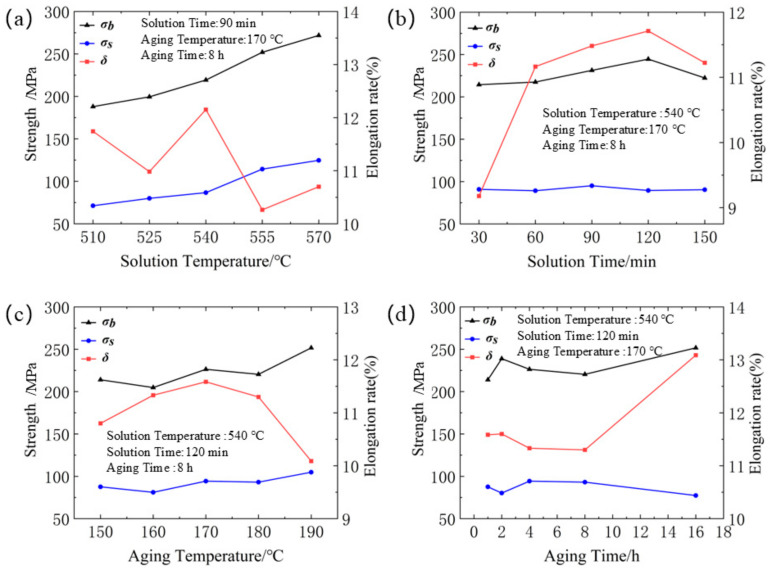
Influence of heat treatment parameters on mechanical behavior: (**a**) solution temperature; (**b**) solution time; (**c**) aging temperature; (**d**) solution time.

Based on the orthogonal experimental design table shown in Table 1, the macro-mechanical property parameters of 6061 aluminum alloy under different heat treatment conditions are illustrated in Figure 9.

**Figure 10:**
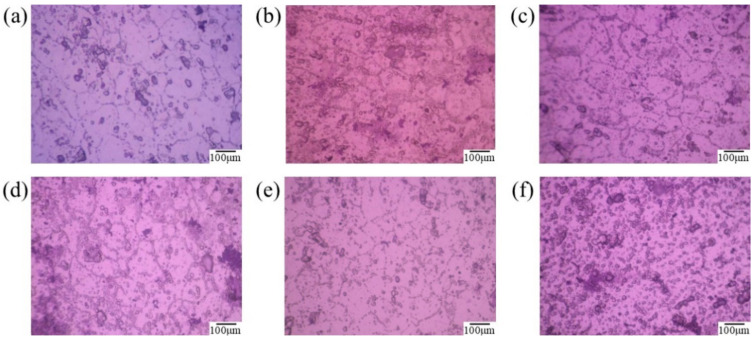
Metallographic structure of 6061 aluminum alloy at different solid solution temperatures: (**a**) Primitive state; (**b**) 510 °C; (**c**) 525 °C; (**d**) 540 °C; (**e**) 555 °C; (**f**) 570 °C. Solution time: 90 min.

The experimental results indicate that the trend of the effect of solution treatment temperature on tensile strength (σ_b_) and yield strength (σ_s_) is similar. As the solution treatment temperature increased from 510 °C to 555 °C, the tensile strength increased by 64.019 MPa, and the yield strength increased by 43.173 MPa. This is attributed to the increased solubility of the alloy with higher solution treatment temperatures, which results in the precipitation of more phases during the aging stage, thereby enhancing the strength of the alloy. However, with further increases in temperature, the rate of increase in tensile strength (σ_b_) and yield strength (σ_s_) slows down. Additionally, when the solution treatment temperature exceeds 540 °C, the elongation at fracture (δ) of the material begins to decrease. Although the precipitated phases can enhance the material’s strength, they also increase the dislocation density at the grain boundaries, which contributes to increased brittleness. The microstructures obtained under different solution treatment temperatures are shown in Figure 10. Among them, the solution time is a constant parameter, set to 90 min. The microscopic characterization results reveal that the heating temperature during solution treatment has a significant impact on the microstructure of 6061 aluminum alloy. Comparison of the microstructures under different solution treatment temperatures shows that recrystallization of the original structure occurs during the solution heating process. As the heating temperature increases, the driving force for recrystallization of the 6061 aluminum alloy gradually increases. The connectivity of grain boundaries improves, and the precipitates in the original structure gradually dissolve, leading to a reduced ability to impede dislocations. Consequently, the dispersion strengthening effect of the secondary phases on the matrix gradually diminishes. At a solution treatment temperature of 540 °C, the recrystallization process of the aluminum alloy is complete, with recrystallized grains sufficiently grown and evenly distributed. Therefore, during the solution treatment of 6061 aluminum alloy, as the solution temperature increases from 510 °C to 540 °C, the alloy’s plasticity gradually improves. However, when the solution temperature is further increased to 570 °C, the grains in the alloy grow through grain boundary coalescence, and a large number of re-dissolved precipitates form at the grain boundaries, increasing boundary brittleness. This leads to a reduction in the macro-mechanical properties of the 6061 alloy.

The effect of solution treatment time on the material’s yield strength, tensile strength, and elongation at fracture is shown in Figure 9b. As the solution treatment time increases, both the tensile strength (σ_b_) and yield strength (σ_s_) exhibit a generally stable trend. This is because the secondary phase particles in the material dissolve sufficiently within a short period, so the impact of solution treatment time on the material’s strength is minimal. Longer solution treatment times help to alleviate stress concentrations at grain boundaries and reduce discrepancies between grains, thereby improving the material’s elongation. However, excessively long solution treatment times may cause grain growth, and excessively large grains can reduce the material’s plasticity, leading to a decrease in elongation. The elongation (δ) generally shows an increasing trend followed by a decrease with extended solution treatment times, reaching a maximum value of 11.71 at 120 min.

The effect of aging temperature on the material’s yield strength, tensile strength, and elongation at fracture is shown in Figure 9c. As the aging temperature increases, the strength of the material generally rises. The tensile strength increases by 37.615 MPa at 190 °C compared to 150 °C, indicating a significant impact of aging temperature on the material’s strength. Higher temperatures facilitate atomic diffusion, accelerate the formation of Guinier–Preston (G.P.) zones, and promote the nucleation and growth of strengthening phases, thereby significantly enhancing the alloy’s strength. At the same time, the elongation of the material is also highly sensitive to the aging temperature, with a sharp decrease in elongation occurring above 170 °C. Specifically, elongation decreases by 12.9% at 190 °C compared to 170 °C. This reduction is attributed to the promotion of nucleation and growth of the primary strengthening phase, T1, at higher temperatures in the 6061 aluminum–lithium alloy. The T1 phase predominantly nucleates at crystal defects such as dislocations, leading to reduced elongation at fracture.

**Figure 11:**
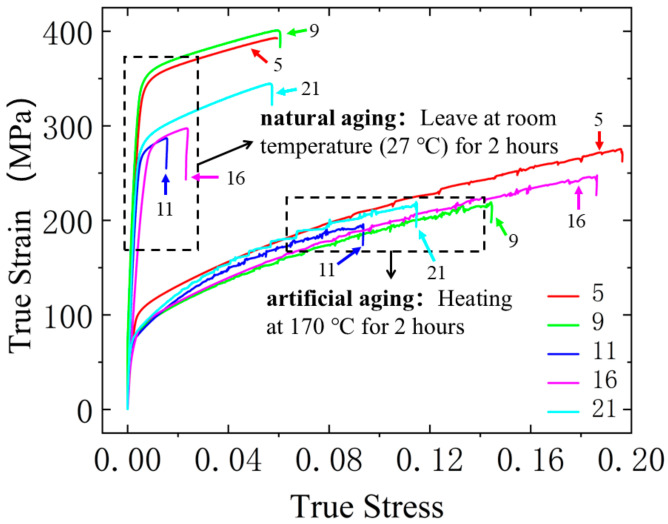
Mechanical behavior of materials under different aging conditions (5 solution treatments): (510 °C × 150 min); 9 (525 °C × 120 min); 11 (540 °C × 30 min); 16 (555 °C × 30 min); 21 (570 °C × 30 min).

Artificial aging accelerates the precipitation of new phases by heating to higher temperatures, allowing for faster precipitation and enabling the control of aging temperature and time to achieve different microstructural and strengthening effects. However, while artificial aging significantly shortens the aging time, it can result in noticeable differences in the strengthening effects on aluminum alloys. To investigate the impact of artificial aging versus natural aging on the strengthening effect of 6061 aluminum alloy, the true stress–strain curves under different aging conditions were obtained, as shown in Figure 11. The experimental results indicate that, under the same solution treatment temperature conditions, the plasticity of 6061 aluminum alloy is significantly superior after artificial aging compared to natural aging, and natural aging notably enhances the alloy’s strength.

**Figure 12:**
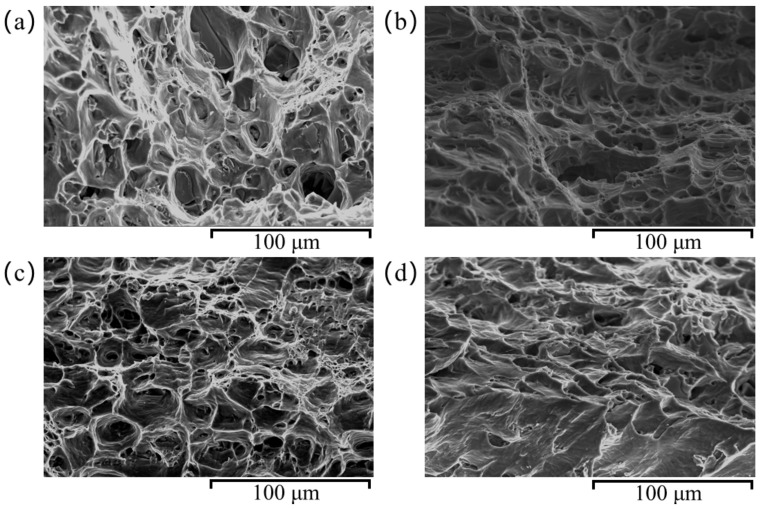
Fracture microstructure under different heat treatment methods: (**a**) 525 °C × 120 min + 150 °C × 16 h; (**b**) 540 °C × 120 min + 150 °C × 16 h; (**c**) 540 °C × 120 min + 170 °C × 16 h; (**d**) 555 °C × 120 min + 170 °C × 16 h.

The quasi-static tensile fracture morphology under four different heat treatment conditions was observed using a Quanta 200 environmental scanning electron microscope (ESEM), as shown in Figure 12.

A comparison between Figure 12a,b shows that, under the same aging conditions, a fracture morphology primarily consists of a few 10 μm diameter dimples and tearing ridges, with relatively shallow dimple depths. This indicates that the 6061 aluminum alloy experiences limited plastic deformation before fracture. As the solution treatment temperature increases to 540 °C, the density, depth, and size of the dimples on the fracture surface gradually increase, with a corresponding rise in plastic fracture characteristics. This suggests that as the solution treatment temperature increases, the material’s plasticity improves. At higher temperatures, the interaction between dislocations becomes more intense, resulting in the formation of more entangled structures and shear bands, which in turn promotes an increase in plastic deformation, thereby enhancing the material’s ductile fracture characteristics. Further comparison of Figure 12b,c reveals that, under the same solution treatment temperature conditions, the distribution of dimples on the fracture surface becomes more uniform as the aging temperature increases. The proportion and depth of large dimples significantly increase, indicating a notable enhancement in plasticity after solution treatment at 540 °C for 120 min and aging at 170 °C for 16 h. The aging process promotes the formation of precipitates at the grain boundaries, which strengthens the boundaries and reduces the movement of grains, thereby improving the material’s resistance to deformation and enhancing its plasticity. As the aging temperature increases, dislocations become more mobile, and grain boundary strengthening becomes more uniform, leading to a more consistent distribution of dimples on the fracture surface. However, when the solution treatment temperature is further increased to 570 °C, as shown in Figure 12d, the fracture surface exhibits distinct cleavage features with extensive river-like patterns and a significant reduction in the number of dimples, indicating a shift toward brittle fracture characteristics. Based on these microscopic observations, combined with macro-mechanical properties, it can be concluded that the 6061 aluminum alloy achieves optimal overall mechanical performance after solution treatment at 540 °C for 120 min, followed by artificial aging at 170 °C for 16 h.

**Figure 13:**
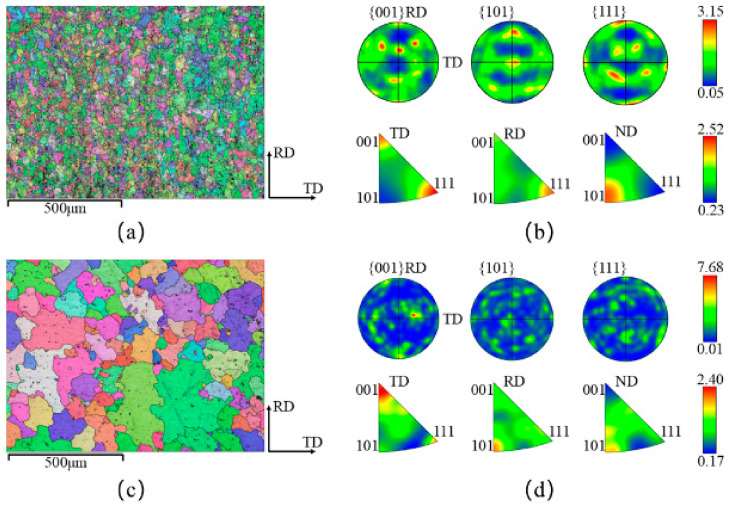
Grain orientation, pole, and reverse pole diagrams: (**a**,**b**) Original state; (**c**,**d**) Solution temperature: 540 °C; solution time: 2 h.

**Figure 14:**
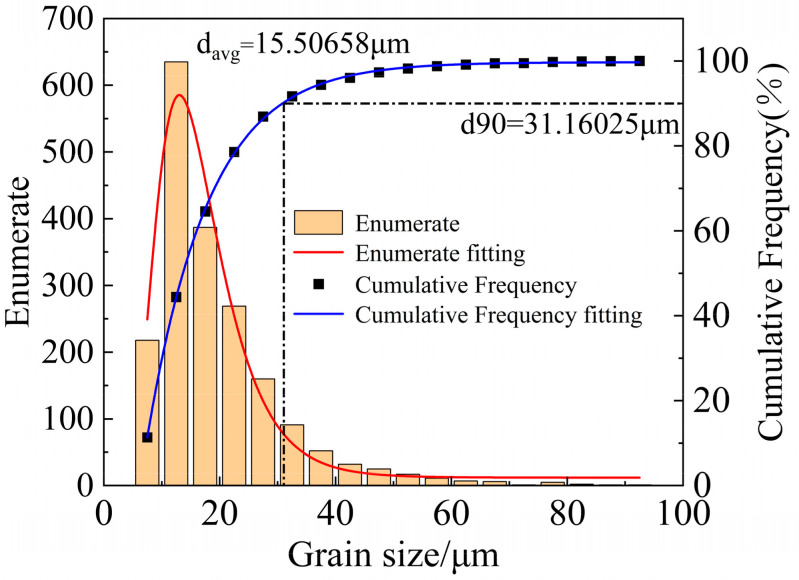
Grain size distribution in the original state.

**Figure 15:**
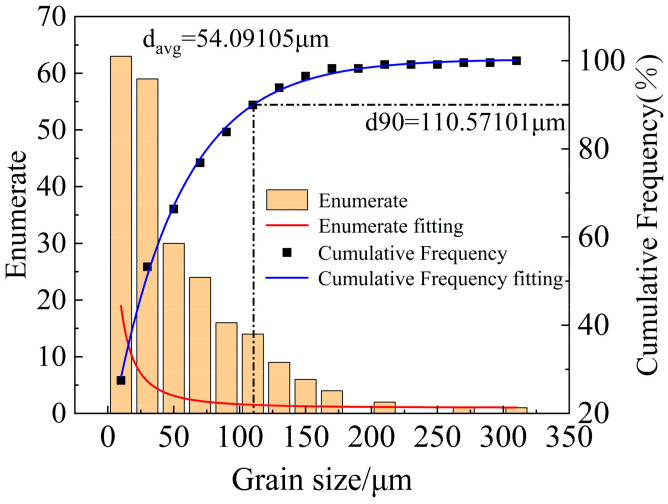
Grain size distribution: 540 °C × 2 h.

Figure 13 shows the grain orientation maps, pole figures, and inverse pole figures of 6061 aluminum alloy in the as-received state and after solution treatment at 540 °C. As shown in Figure 13a, the grains in the as-received 6061 aluminum alloy are unevenly distributed, with noticeable fragmented grains, and the grain colors vary. The grain color reflects the grain orientation, indicating a random distribution of grain orientations in the as-received state. In contrast, after solution treatment at 540 °C, as shown in Figure 13c, the grain size of 6061 aluminum alloy has significantly increased, and smaller grains are observed at the grain boundaries. Additionally, the grain orientations have tended to align more uniformly. According to the grain size distribution histogram shown in Figure 14 and Figure 15, the average grain size in the as-received sample is 15.5 μm. The cumulative frequency distribution curve reveals that approximately 90% of the grains have sizes smaller than 31.2 μm. After solution treatment at 540 °C, the average grain size increases to 54.1 μm, with the cumulative frequency distribution curve indicating that about 90% of the grains are smaller than 110.6 μm, and very few grains exceed 200 μm. Comparing the pole figures and inverse pole figures shown in Figure 13b,d, it is evident that the maximum pole density of the texture in the as-received 6061 aluminum alloy is 3.15 mrd, with a random distribution of texture without any distinct preferred orientation. After solution treatment at 540 °C, the maximum pole density increases to 7.68 mrd, showing a strong {001} texture. This result further confirms that the grain orientations in the as-received 6061 aluminum alloy are randomly distributed, whereas solution treatment induces a more pronounced preferred orientation in the alloy.

## 4. Constitutive Model

The Hansel–Spittel model comprehensively considers the effects of temperature, strain rate, and strain on stress. It is widely applicable, relatively simple in form, and convenient to use [22].

Analyzing the true stress–true strain curve for 6061 aluminum alloy reveals that flow stress is influenced not only by strain and heat treatment temperature but also by strain rate. In this study, the Hansel–Spittel model is employed to describe the hot deformation behavior of 6061 aluminum alloy. The model’s equation is given by the following equation:

(1) $$\sigma =Ae^{m_{1}T}\varepsilon^{m_{2}}{\dot{\varepsilon }}^{m_{3}}e^{\frac{m_{4}}{\varepsilon }}(1+\varepsilon {)}^{m_{5}T}e^{m_{7}\varepsilon }{{\dot{\varepsilon }}^{m_{8}}}^{T}T^{m_{9}}$$

where σ is the flow stress, *A* is a material constant, e is the base of the natural logarithm, T is the deformation temperature, ε is the strain, $\overset{˙}{\varepsilon }$ is the strain rate, *m*_1_ is the temperature-related coefficient, *m*_2_ is the strain hardening coefficient, *m*_3_ is the strain rate hardening coefficient, *m*_4_ is the strain softening coefficient, *m*_5_ is the temperature-dependent strain hardening coefficient, *m*_7_ is the strain-related coefficient, *m*_8_ is the temperature-dependent strain rate hardening coefficient, *m*_9_ is the temperature coefficient.

Taking the natural logarithm of both sides of Equation (1) yields the following:

(2) $$ln\sigma =lnA+m_{1}T+m_{2}ln\varepsilon +m_{3}ln\dot{\varepsilon }+\frac{m_{4}}{\varepsilon }+m_{5}Tln\left(1+\varepsilon \right)+m_{7}\varepsilon +m_{8}Tln\dot{\varepsilon }+m_{9}lnT$$

### 4.1. Fitting of m_3_ and m_8_ Values

When the temperature and strain (*T*, ε) are constant, $lnA+m_{1}T+m_{2}ln\varepsilon +m_{4}/\varepsilon +m_{9}lnT$ + $m_{5}Tln\left(1+\varepsilon \right)+m_{7}\varepsilon$ in Equation (2) can be regarded as a constant *K*_1_. Therefore, Equation (2) can be transformed into the following:

(3) $$ln\sigma =ln\dot{\varepsilon }\left(m_{3}+m_{8}T\right)+K_{1}$$

**Figure 16:**
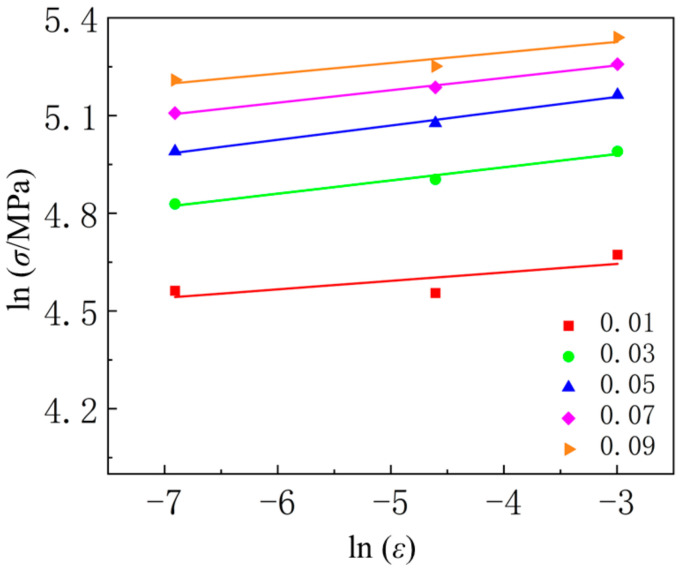
The relationship between lnσ and $ln\dot{\varepsilon }$ under different strains at 540 °C.

Plot the experimental data under different conditions with coordinates lnσ and $ln\dot{\varepsilon }$, taking the solid solution temperature of 540 °C as an example, as shown in Figure 16.

**Figure 17:**
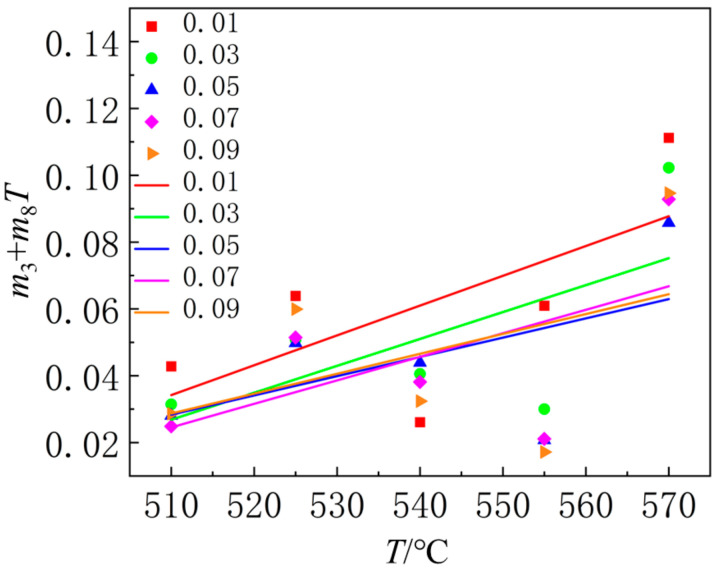
The relationship between *m*_3_ + *m*_8_*T* and *T* under different strains at 540 °C.

From the figure, it can be observed that the relationship between lnσ and $ln\dot{\varepsilon }$ is linear, with the slope of the line representing the value of *m*_3_ + *m*_8_*T* under different strain conditions. The relationship between *m*_3_ + *m*_8_*T* and *T* for various strains is shown in Figure 17. The figure indicates that *m*_3_ + *m*_8_*T* varies linearly with *T* under different strain conditions. The intercept and slope of the line correspond to the values of *m*_3_ and *m*_8_, respectively. By calculating the arithmetic average of *m*_3_ and *m*_8_, the values obtained are *m*_3_ = 0.353852 and *m*_8_ = 0.000742067.

### 4.2. Fitting of m_1_, m_5_, m_9_ Values

When the strain and strain rate (i.e., ε, $\dot{\varepsilon }$) are constant, $lnA+m_{2}ln\varepsilon +m_{3}ln\dot{\varepsilon }+m_{4}/\varepsilon +m_{7}\varepsilon +m_{8}Tln\dot{\varepsilon }$ can be regarded as a constant. Let it be *K*_2_, then Equation (2) can be transformed into the following:

(4) $$ln\sigma =K_{2}+[m_{1}+m_{8}ln\dot{\varepsilon }+m_{5}\ln\left(1+\varepsilon \right)]T+m_{9}lnT$$

**Figure 18:**
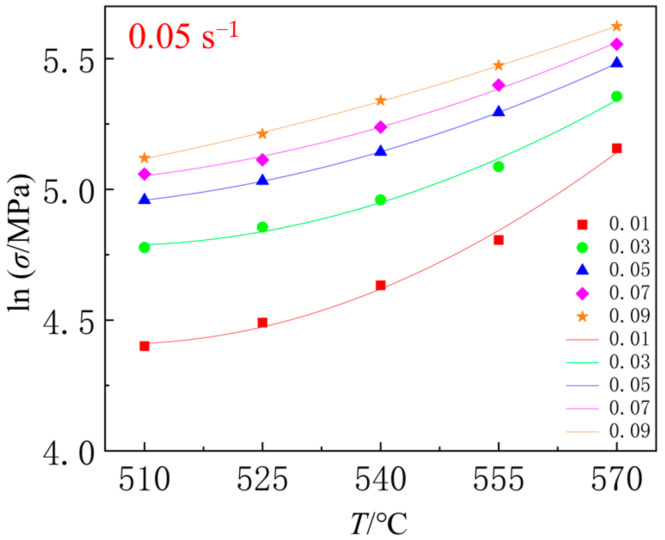
The relationship between lnσ and *T* at the same strain rate.

Using the Origin software (2022), the relationship between lnσ and *T* at the same strain rate was fitted with $y=kx+m_{9}lnx+K_{2}$. Taking the strain rate of 0.05 s^−1^ as an example, as shown in Figure 18, the values of *m*_9_ and *k* at strain rates of 0.001 s^−1^ and 0.01 s^−1^ can also be obtained through fitting. The average value of *m*_9_ for different conditions is *m*_9_ = 0.964302. From Equation (4), it can be seen that $k=m_{1}+m_{8}ln\dot{\varepsilon }+m_{5}ln(1+\varepsilon )$. At a certain strain rate, $ln\dot{\varepsilon }$ is a constant value, and $m_{1}+m_{8}ln\dot{\varepsilon }$ is a constant value *K*_3_. Therefore, the value of *k* can be fitted using the function $y=K_{3}+m_{5}ln(1+x)$. By fitting the data using software, the values of *m*_1_ and *m*_5_ can be determined. Averaging the values of *m*_1_ and *m*_5_ under different strain conditions yields *m*_1_ = 0.004321968 and *m*_5_ = −0.011753333.

### 4.3. Fitting of m_2_, m_4_, m_7_ Values

When the temperature and strain rate are constant, $lnA+m_{1}T+m_{3}ln\dot{\varepsilon }+m_{8}Tln\dot{\varepsilon }+m_{9}lnT$ can be regarded as a constant. Let it be *K*_4_, then Equation (2) can be transformed into the following:

(5) $$ln\sigma =K_{4}+m_{2}ln\varepsilon +\left(\frac{m_{4}}{\varepsilon }+m_{7}\varepsilon \right)+m_{5}Tln\left(1+\varepsilon \right)$$

**Figure 19:**
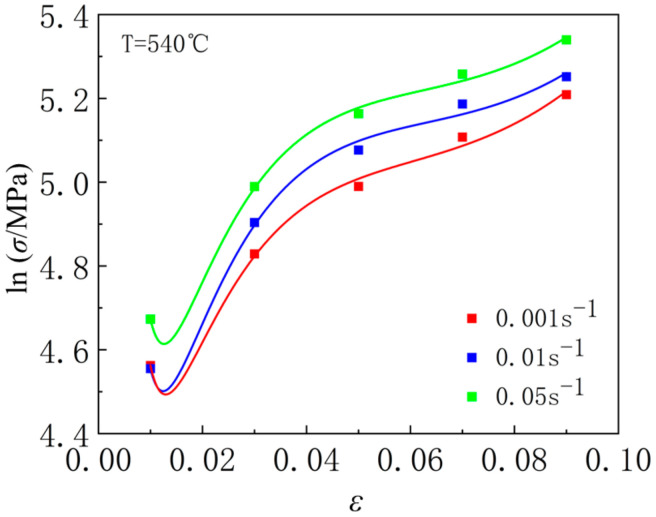
The relationship between lnσ and ε at 540 °C.

At the same temperature, use Origin software to fit the relationship between lnσ and ε at different strain rates using function $y=K_{4}+m_{2}lnx+\left(m_{4}/\varepsilon +m_{7}x\right)+m_{5}ln(1+x)$. Taking 540 °C as an example, as shown in Figure 19. Similarly, create a relationship diagram between the two at other temperatures, fit the values of *m*_2_, *m*_4_, and *m*_7_ at all temperatures, and take the average to obtain *m*_2_ = 1.012090667, *m*_4_ = 0.037345999, *m*_7_ = −0.770635.

### 4.4. Calculate the Value of A

By substituting the previously obtained *m*_1_, *m*_2_, *m*_3_, *m*_4_, *m*_5_, *m*_6_, *m*_7_, *m*_8_, and *m*_9_ into Equation (1), the value of *A* can be calculated. The average value of *A* is *A* = 1.36630552. By substituting these parameter values into Equation (1), the tensile constitutive equation of 6061 aluminum alloy is obtained as follows:

(6) $$\sigma =1.36630552e^{0.004321968T}\varepsilon^{1.012090667}{\dot{\varepsilon }}^{-0.353852}e^{\frac{0.037345999}{\varepsilon }}{\left(1+\varepsilon \right)}^{-0.011753333T}e^{-0.770635\varepsilon }{\dot{\varepsilon }}^{0.000742067T}T^{0.964302}$$

### 4.5. Verification of Constitutive Equation

**Figure 20:**
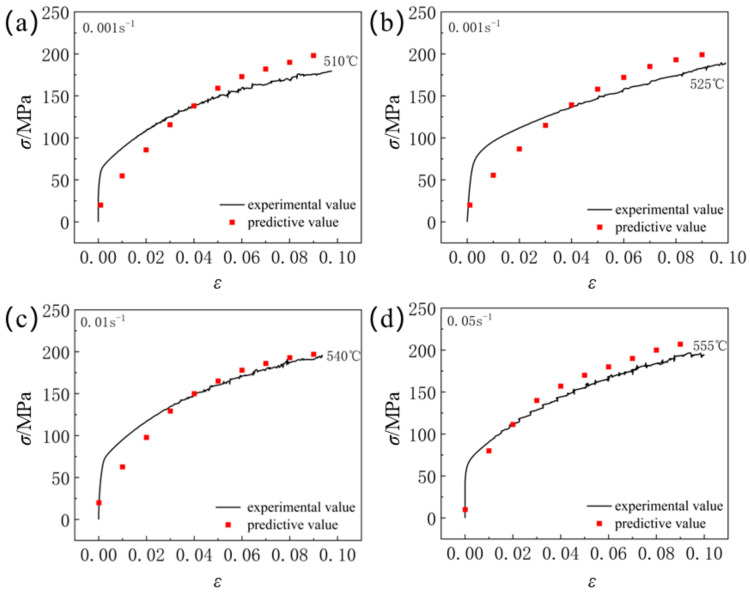
Comparison of rheological stress test values and calculated values under different deformation conditions: (**a**) 510 °C × 2 h + 180 °C × 8 h; (**b**) 525 °C × 90 min + 180 °C × 8 h; (**c**) 540 °C × 2 h + 150 °C × 1 h; (**d**) 555 °C × 2 h + 160 °C × 2 h.

By substituting various deformation parameters such as temperature, strain, and strain rate into Equation (6), the deformation stress of the material can be predicted. The stress values obtained from Equation (6) compared with experimental stress values are shown in Figure 20. It is observed that the model effectively describes the deformation characteristics of 6061 aluminum alloy.

Figure 20 can only qualitatively show the relationship between predicted values and experimental values. To quantitatively describe the accuracy of Equation (6), the correlation coefficient (*R*) and average relative error (*AARE*) in statistics can be used for verification [10]. The formula is expressed as follows:

(7) $$R=\frac{\sum_{i=1}^{N}\left(E_{i}-\bar{E}\right)\left(P_{i}-\bar{P}\right)}{\sqrt{\sum_{i=1}^{N}{\left(E_{i}-\bar{E}\right)}^{2}\sum_{i=1}^{N}{\left(P_{i}-\bar{P}\right)}^{2}}}$$

(8) $$AARE\left(\%\right)=\frac{1}{N}\sum_{i=1}^{N}\left|\frac{E_{i}-P_{i}}{E_{i}}\right|\times 100$$

In this context, E represents the experimental results, and P denotes the predicted values obtained from the constitutive equations. The variables $\bar{E}$ and $\bar{P}$ are the averages of *E* and *P*, respectively. *N* denotes the number of data points used in the survey. The correlation coefficient *R* is a commonly used statistical parameter that provides information on the strength of the linear relationship between the measured and calculated values. The Average Absolute Relative Error (*AARE*) is calculated through the relative errors of individual comparisons and thus serves as an unbiased statistical parameter for assessing the predictive capability of the equation.

**Figure 21:**
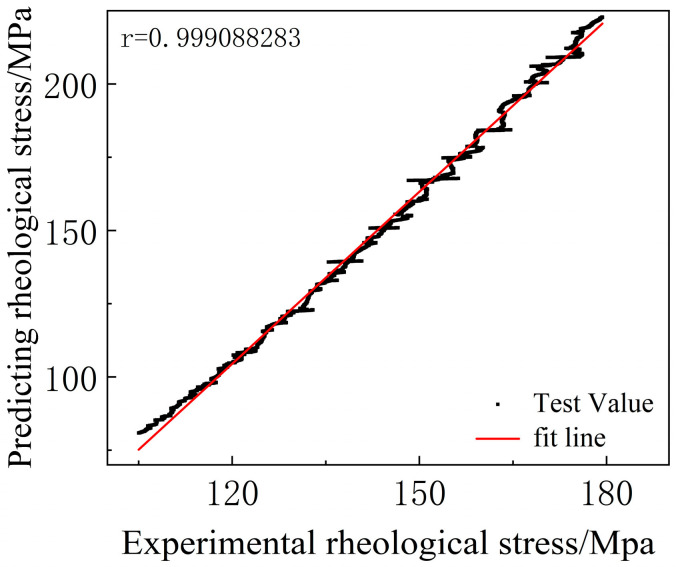
The relationship between rheological stress test values and predicted values under different deformation conditions.

As shown in Figure 21, the correlation coefficient *R* between the experimental values and the predicted values is 0.999088283, indicating a strong linear correlation. The Average Absolute Relative Error (*AARE*) is 13.42375564%, reflecting good predictive capability. The correlation coefficient *R* suggests that the Hansel–Spittel model established in this study can accurately predict the deformation characteristics of 6061 aluminum alloy.

## 5. Conclusions

(1) Commercial cold-rolled 6061 aluminum plates exhibit significant anisotropy. The mechanical properties along the rolling direction (RD) and in the direction 90° to the rolling direction (LT) are significantly superior to those in the 45° direction relative to the rolling direction. During the plastic deformation process, both the yield strength and tensile strength of 6061 aluminum alloy decrease with increasing strain rate, while the plastic elongation shows a decreasing trend as the strain rate increases.
(2) Within the temperature range of 510 °C to 540 °C, the plasticity of 6061 aluminum alloy gradually increases while the hardness decreases. When the solution treatment temperature is raised to 570 °C, the grain size of the 6061 aluminum alloy significantly increases, the brittleness of the grain boundaries rises, and the plasticity decreases. The effect of solution treatment time on the material’s strength is relatively small. As the aging temperature increases, the overall strength of the material shows a rising trend. The elongation of the 6061 aluminum alloy first decreases and then increases with the aging time.
(3) Based on the experimental results, a Hansel–Spittel rheological stress model for 6061 aluminum alloy was constructed. The correlation coefficient (*R*) between the predicted results of the model and the experimental measurements is 0.9991, indicating a strong linear correlation. The Average Absolute Relative Error (*AARE*) is 13.42%, demonstrating that the established Hansel–Spittel rheological stress model can accurately predict the rheological stress characteristics of 6061 aluminum alloy.

The strain rate effect observed in this study is quite interesting, but due to limited supporting data to explain this unique phenomenon, the cause of this effect is still under investigation. Efforts are being made to explain it from a microscopic perspective. However, based on the current research, a suitable explanation for this phenomenon has not yet been found. We will continue to explore this phenomenon, as it is the next focal point of our research.

## Figures and Tables

**Table 1:** Chemical composition table of 6061 aluminum alloy (wt.%).

Mg	Si	Mn	Fe	Cu	Cr	Zn	Ti	Al
0.97	0.13	0.12	0.56	0.27	0.12	0.08	0.023	Balance

**Table 2:** Orthogonal experimental factors and levels for heat treatment.

Level	A. Solution Temperature /°C	B. Solution Time /min	C. Aging Temperature /°C	D. Aging Time /h
1	510	30	150	1
2	525	60	160	2
3	540	90	170	4
4	555	120	180	8
5	570	150	190	16

**Table 3:** Mechanical performance parameters of tensile test specimens.

Strain Rate (s^−1^)	Direction	Yield Strength σ_s_ (MPa)	Tensile Strength σ_b_ (MPa)	Elongation Rate Δ (%)
0.05	RD	347	395	9.0
	ST	306	355	7.9
	LT	321	378	9.8
0.01	RD	346	395	10.9
	ST	312	357	7.4
	LT	334	378	10.5
0.001	RD	345	391	10.4
	ST	345	355	7.4
	LT	310	367	8.2

## Data Availability

The raw data supporting the conclusions of this article will be made available by the authors on request.
